# Evaluation of the use of methylprednisolone and dexamethasone in asthma critically ill patients with COVID-19: a multicenter cohort study

**DOI:** 10.1186/s12890-023-02603-4

**Published:** 2023-08-28

**Authors:** Khalid Al Sulaiman, Ohoud Aljuhani, Ghazwa B. Korayem, Ali Altebainawi, Reham Alharbi, Maha Assadoon, Ramesh Vishwakarma, Nadia H. Ismail, Asma A. Alshehri, Faisal E. Al Mutairi, Mashael AlFaifi, Abdullah F. Alharthi, Abeer A. Alenazi, Mai Alalawi, Omar Al Zumai, Hussain Al Haji, Sarah T. Al Dughaish, Abdulrahman S. Alawaji, Haifa A. Alhaidal, Ghassan Al Ghamdi

**Affiliations:** 1 Pharmaceutical Care Department, King Abdulaziz Medical City (KAMC), Riyadh, Saudi Arabia; 2 College of Pharmacy, King Saud Bin Abdulaziz University for Health Sciences (KSAU-HS), Riyadh, Saudi Arabia; 3 King Abdullah International Medical Research Center (KAIMRC), KSAU-HS, PO Box 22490, Riyadh, 11426 Saudi Arabia; 4 Saudi Critical Care Pharmacy Research (SCAPE) Platform, Riyadh, Saudi Arabia; 5 Department of Pharmacy Practice, Faculty of Pharmacy, King Abdulaziz University, Jeddah, Saudi Arabia; 6 Department of Pharmacy Practice, Princess Nourah bint Abdulrahman University, Riyadh, P.O.Box 84428, 11671 Saudi Arabia; 7 Pharmaceutical Care Services, King Khalid Hospital, Hail, Saudi Arabia; 8 College of Pharmacy, University of Hail, Hail, Saudi Arabia; 9 Norwich Medical School, University of East Anglia, Norwich, UK; 10 King Fahad Hospital of the University, AL-Khobar, Saudi Arabia; 11 Department of Pharmacy Practice, College of Clinical Pharmacy, Imam Abdulrahman Bin Faisal University, Dammam, Saudi Arabia; 12 Pharmaceutical Care Department, Prince Sultan Military Medical City, Riyadh, Saudi Arabia; 13 Pharmaceutical Services Administration, King Saud Medical City, Riyadh, Saudi Arabia; 14 College of Pharmacy, Shaqra University, Shaqra, Saudi Arabia; 15 Pharmaceutical Care Department, King Abdulaziz Medical City, Jeddah, Saudi Arabia; 16 Respiratory Department Services, King Abdulaziz Medical City (KAMC), Riyadh, Saudi Arabia; 17 College of Applied Medical Sciences, King Saud Bin Abdulaziz University for Health Sciences (KSAU-HS), Riyadh, Saudi Arabia; 18 Pharmaceutical Care Department, King Khalid Eye Specialist Hospital, Riyadh, Saudi Arabia; 19 College of Medicine, King Saud Bin Abdulaziz University for Health Sciences, Riyadh, Saudi Arabia; 20 Intensive Care Department, King Abdulaziz Medical City, Riyadh, Saudi Arabia

**Keywords:** Asthma, COVID-19, SARS-CoV-2, Dexamethasone, Methylprednisolone, Mortality, MV duration, Length of stay (LOS), Critically ill, Intensive Care Units

## Abstract

**Background:**

Previous studies have shown mortality benefits with corticosteroids in Coronavirus disease-19 (COVID-19). However, there is inconsistency regarding the use of methylprednisolone over dexamethasone in COVID-19, and this has not been extensively evaluated in patients with a history of asthma. This study aims to investigate and compare the effectiveness and safety of methylprednisolone and dexamethasone in critically ill patients with asthma and COVID-19.

**Methods:**

The primary endpoint was the in-hospital mortality. Other endpoints include 30-day mortality, respiratory failure requiring mechanical ventilation (MV), acute kidney injury (AKI), acute liver injury, length of stay (LOS), ventilator-free days (VFDs), and hospital-acquired infections. Propensity score (PS) matching, and regression analyses were used.

**Results:**

A total of one hundred-five patients were included. Thirty patients received methylprednisolone, whereas seventy-five patients received dexamethasone. After PS matching (1:1 ratio), patients who received methylprednisolone had higher but insignificant in-hospital mortality in both crude and logistic regression analysis, [(35.0% vs. 18.2%, *P* = 0.22) and (OR 2.31; CI: 0.56 – 9.59; P = 0.25), respectively]. There were no statistically significant differences in the 30-day mortality, respiratory failure requiring MV, AKI, acute liver injury, ICU LOS, hospital LOS, and hospital-acquired infections.

**Conclusions:**

Methylprednisolone in COVID-19 patients with asthma may lead to increased in-hospital mortality and shorter VFDs compared to dexamethasone; however, it failed to reach statistical significance. Therefore, it is necessary to interpret these data cautiously, and further large-scale randomized clinical trials are needed to establish more conclusive evidence and support these conclusions.

**Supplementary Information:**

The online version contains supplementary material available at 10.1186/s12890-023-02603-4.

## Introduction

The symptoms experienced by patients infected with Coronavirus disease-19 (COVID-19) may vary from mild to severe which may lead to death [1]. The symptoms are mainly characterized by mild upper respiratory symptoms, fever, fatigue, pneumonia, and acute respiratory syndrome [2]. The primary cause of mortality among patients with COVID-19 is respiratory complications [3]. Moreover, patients with COVID-19 and pre-existing comorbid diseases have worse disease outcomes, including increased incidence of hospitalization, intensive care unit (ICU) admission, and mortality [4]. This risk increases in patients with asthma [5–7]. Since patients with asthma experience immune system imbalance which leads to a chronic inflammatory condition of the airways [8].

In critically ill patients, severe COVID-19 can increase the systemic inflammatory response, resulting in a systemic hyperinflammatory state that can cause a range of complications [1]. Therefore, corticosteroids have been utilized to treat patients with severe acute respiratory distress syndrome (ARDS) due to their ability to modulate the immune system. There have been several studies that have proven the benefit of corticosteroid use in patients with COVID-19 [9–11]. A retrospective study that assessed 201 patients with COVID-19 found that the use of methylprednisolone significantly reduced mortality (HR, 0.38; 95% CI, 0.20–0.72) [9]. Moreover, a meta-analysis including 20,197 patients with COVID-19 from 44 studies, found that the corticosteroids group experienced a substantial decrease in mortality rate [10].

Several studies compared the use of both dexamethasone and methylprednisolone in patients infected with COVID-19 [12–14]. In a prospective study, the efficacy of dexamethasone and methylprednisolone in moderate to severe COVID-19 disease was found to be equal [12].Another study compared the efficacy of methylprednisolone versus dexamethasone in hospitalized patients with COVID-19 and reported that methylprednisolone led to a shorter hospital stay, less need for mechanical ventilation, and better clinical status on days 5 and 10 [13]. In addition, methylprednisolone and dexamethasone were compared in a retrospective study in ICU patients with COVID-19 [14]. The study found that methylprednisolone caused a greater reduction in mortality in patients with COVID-19 who needed mechanical ventilation when compared to dexamethasone [14].

As far as we know, few of these previous studies indicated if any patients had a history of asthma along with COVID-19 [12–14]. Meanwhile, there is inconsistency about the benefit of methylprednisolone over dexamethasone in patients with COVID-19 [12–14]. The use of systemic corticosteroids is more essential in patients with asthma. However, the benefit of one systemic corticosteroid agent over another in patients with asthma and COVID-19 has not been extensively evaluated. Therefore, this study aims to compare the effectiveness and safety of methylprednisolone and dexamethasone use in critically ill patients with COVID-19 and asthma.

## Methods

### Study design

This study is part of the Saudi Critical Care Pharmacy Research (SCAPE) platform, which conducted several observational studies that evaluate the safety and effectiveness of multiple therapies in critically ill patients [1, 15–20]. This study was a multi-center, retrospective cohort study in adult critically ill COVID-19 patients with known asthma who were admitted to the ICU in five Saudi Arabian hospitals between March 1, 2020, and July 31, 2021. Eligible patients were categorized based on the type of corticosteroid therapy used during their ICU stay into dexamethasone or methylprednisolone. Reverse Transcriptase-Polymerase Chain Reaction (RT-PCR) nasopharyngeal or throat swabs were used to confirm the COVID-19 diagnosis. Asthma as a comorbidity was labeled based on the patient’s chart and documentation using the electronic medical record. All patients were followed until they were discharged or died during the in-hospital stay. The study protocol was reviewed and approved by the Institutional Review Board (IRB) at King Abdullah International Medical Research Center (KAIMRC), Riyadh, Saudi Arabia (Ref.# NRC23R-049–02).

### Study setting

The research was carried out at five medical facilities located in Saudi Arabia. Included centers were specifically chosen because they had advanced intensive care unit (ICU) facilities and the capability to handle critically ill patients diagnosed with COVID-19 effectively. Moreover, these centers adhered to the standardized national COVID-19 management protocol established by the Ministry of Health (MOH).

The selection criteria for these centers included various factors such as their geographic distribution, ensuring representation from different regions within Saudi Arabia. Additionally, the availability of electronic medical records was considered, to facilitate efficient data collection and analysis for the study. Finally, the willingness of the medical centers to actively participate in the research was also taken into account, ensuring their commitment and cooperation throughout the study; details of participating hospitals and the leading centers can be found in the Supplementary file 1.

### Study participants

**Fig. 1:**
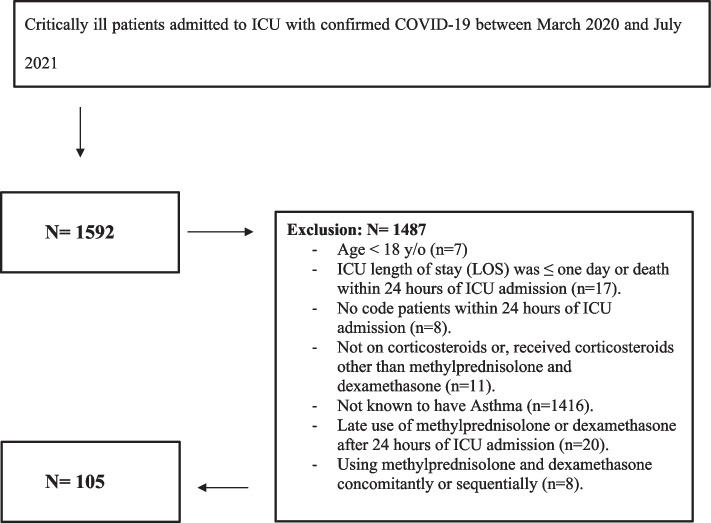
Eligibility criteria flowchart

All COVID-19 critically ill adult patients (age ≥ 18 years) who were admitted to the ICUs in the above centers and within the mentioned period were assessed for eligibility. Patients were excluded if they did not receive either dexamethasone or methylprednisolone, were not known to have asthma, received corticosteroids after 24 h of ICU admission, used methylprednisolone and dexamethasone concurrently or sequentially, died within the first 24 h of ICU admission, ICU length of stay (LOS) ≤ one day, or were labeled as "Do-Not-Resuscitate" within 24 h of ICU admission (Fig. 1).

### Data collection

A comprehensive compilation of medical records numbers (MRNs) for critically ill patients admitted to the intensive care unit (ICU) with COVID-19 was obtained from the electronic health records of the collaborating medical facilities. Subsequently, the patient's eligibility was evaluated based on predetermined eligibility criteria. Following this assessment, a dedicated team of co-investigators from each center was responsible for entering the gathered data into the Research Electronic Data Capture (REDCap®) platform.

To ensure the accuracy and consistency of the collected information, the research team leader at each participating center regularly reviewed all the data. The REDCap® platform was utilized for the collection of various variables and data, encompassing demographic information, comorbidities, baseline severity scores (e.g., APACHE II, SOFA), laboratory baseline (e.g., serum creatinine, lactic acid, PaO_2_/FiO_2_ ratio, inflammatory biomarkers); details of data collected could be found in Supplementary file 1.

### Endpoint (s)

The primary endpoint was the in-hospital mortality. Secondary endpoints include 30-day mortality, respiratory failure requiring MV, AKI, acute liver injury, ICU LOS, hospital LOS, ventilator-free days (VFDs), and hospital-acquired infections (bacterial/fungal) (Supplementary file 1).

### Statistical analysis

Patients who received dexamethasone therapy were matched to patients who received methylprednisolone using Propensity score (PS) matching procedure (Proc PS match). Greedy nearest-neighbor matching method was used based on patient’s age, baseline SOFA score, hypertension as a comorbidity, higher baseline FiO_2_ requirement, platelets count, and use of Tocilizumab within 24 h of ICU admission.

One dexamethasone-treated patient was paired with one methylprednisolone-treated patient (1:1 ratio). Patients were matched only if the difference in the logits of the PS for pairs of patients from the two groups was less than or equal to 0.1 times the pooled estimate of the standard deviation (SD). We considered a *P*-value of < 0.05 to be statistically significant. The SAS software was used for all statistical analyses (SAS Version 9.4, SAS Institute Inc. Cary, NC, USA) (Supplementary file 1).

## Results

Among 1592 critically ill patients with COVID-19 who were initially screened, one hundred-five patients with known asthma were included (Fig. 1). Methylprednisolone was given to thirty patients, whereas seventy-five received dexamethasone. Table 1 summarizes the baseline characteristics before and after PS matching in patients who received methylprednisolone compared with dexamethasone. Before PS matching, patients who received methylprednisolone were younger (59.8 ± 13.7 vs. 65.9 ± 13.8), had a lower A-A gradient, oxygenation index, platelets count, alanine transaminase (ALT), and CRP. On the other hand, patients in the control group have a lower PaO2/FiO2 ratio, INR, and aspartate transaminase (AST). However, in post-PS matching, the two groups were comparable, except for gender and baseline blood glucose level (Table 1).

**Table 1:** Baseline characteristic before and after propensity score matching

	**Before propensity score (PS)**				**After propensity score (PS)**			
	**Overall (*N* = 105)**	**DEX** **(*N* = 75)**	**mPRED** **(*N* = 30)**	***P*-value**	**Overall (*N* = 48)**	**DEX** **(*N* = 24)**	**mPRED** **(*N* = 24)**	***P*-value**
**Age (Years), Mean (SD)**	64.1 (14.02)	65.9 (13.84)	59.8 (13.73)	0.0462*	60.5 (13.56)	60.9 (13.84)	60.0 (13.55)	0.8177*
**Gender – Male, n (%)**	39 ( 37.9)	29 ( 39.7)	10 ( 33.3)	0.5434^^	22 ( 45.8)	15 ( 62.5)	7 ( 29.2)	0.0205^^
**BMI, Median (Q1,Q3)**	31.8 (28.76, 37.75)	31.5 (28.73, 37.63)	32.6 (29.39, 37.75)	0.8970^	31.2 (28.37, 37.75)	30.4 (27.58, 39.32)	31.8 (29.43, 35.51)	0.9830^
**APACHE II score, Median (Q1,Q3)**	12.0 (9.00, 19.00)	12.0 (9.00, 19.00)	12.0 (7.00, 18.00)	0.5583^	12.0 (9.50, 16.00)	11.5 (9.00, 13.00)	12.0 (10.00, 17.50)	0.5765^
**SOFA score, Median (Q1,Q3)**	4.0 (2.00, 7.00)	4.0 (2.00, 7.00)	5.0 (2.00, 7.00)	0.6370^	4.5 (2.00, 7.00)	3.5 (1.50, 7.00)	5.0 (2.00, 6.50)	0.5059^
**Multiple Organ Dysfunction Score, Median (Q1,Q3)**	5.0 (4.00, 6.00)	5.0 (4.00, 6.00)	5.0 (3.00, 6.00)	0.9657^	5.0 (4.00, 6.00)	4.0 (4.00, 5.00)	5.0 (3.00, 6.00)	0.1313^
**Early use of Tocilizumab within 24 h, n (%)**	29 ( 27.9)	24 ( 32.4)	5 ( 16.7)	0.1043^^	9 ( 18.8)	5 ( 20.8)	4 ( 16.7)	0.7115**
**Serum creatinine (mmol/L) baseline, Median (Q1,Q3)**	72.0 (61.00, 97.00)	75.0 (63.00, 99.00)	69.0 (57.00, 87.00)	0.1376^	71.0 (59.00, 87.00)	75.0 (58.00, 92.00)	69.0 (59.00, 85.50)	0.5726^
**Blood Urea nitrogen (BUN) baseline (mmol/L), Median (Q1,Q3)**	5.7 (4.10, 8.30)	5.7 (4.25, 8.25)	5.7 (4.10, 9.90)	0.8170^	5.2 (4.05, 7.70)	4.7 (4.05, 7.45)	5.6 (4.05, 8.70)	0.6133^
**Acute Kidney Injury (AKI) Within 24 h of ICU admission, n (%)**	23 ( 22.1)	17 ( 23.0)	6 ( 20.0)	0.7407^^	9 ( 18.8)	4 ( 16.7)	5 ( 20.8)	0.7115**
**Mechanical Ventilation within 24 h of ICU admission, n (%)**	78 ( 75.0)	53 ( 71.6)	25 ( 83.3)	0.2114^^	39 ( 81.3)	19 ( 79.2)	20 ( 83.3)	0.7115**
**A-A Gradient baseline, Mean (SD)**	386.0 (173.85)	407.1 (164.39)	334.4 (188.40)	0.0547^	358.8 (172.19)	356.2 (160.50)	361.5 (187.21)	0.9179*
**Oxygenation Index (OI) baseline, Mean (SD)**	19.3 (20.19)	25.8 (26.71)	12.7 (7.23)	0.0757^	14.0 (7.66)	15.6 (4.99)	13.5 (8.54)	0.7574*
**Inotropes/vasopressors use within 24 h of admission), n(%)**	22 ( 21.6)	18 ( 25.0)	4 ( 13.3)	0.1918^^	9 ( 18.8)	6 ( 25.0)	3 ( 12.5)	0.2673**
**Lactic acid baseline (mmol/l), Mean (SD)**	1.8 (0.95)	1.8 (0.96)	1.8 (0.93)	0.7279^	1.7 (0.79)	1.8 (0.80)	1.6 (0.79)	0.5080*
**Platelets count baseline (10**^**9**^**/l), Median (Q1,Q3)**	249.5 (202.00, 315.50)	255.0 (208.00, 324.00)	221.0 (190.00, 282.00)	0.0510^	222.0 (201.50, 289.00)	228.0 (205.00, 294.50)	221.0 (191.50, 286.00)	0.5498^
**Total WBC baseline (10**^**9**^**/l), Median (Q1,Q3)**	8.7 (6.66, 11.85)	9.2 (6.83, 12.00)	8.1 (5.72, 10.40)	0.1618^	8.1 (5.86, 10.40)	8.8 (5.59, 10.70)	7.8 (5.86, 9.95)	0.5777^
**International normalized ratio (INR), Median (Q1,Q3)**	1.0 (0.99, 1.14)	1.0 (0.99, 1.08)	1.1 (1.00, 1.16)	0.0729^	1.1 (1.00, 1.15)	1.0 (1.00, 1.09)	1.1 (1.01, 1.16)	0.1318^
**Activated partial thromboplastin time (aPTT) baseline (Seconds), Median (Q1,Q3)**	28.9 (25.60, 31.50)	29.2 (25.60, 32.10)	28.0 (25.60, 31.30)	0.6397^	28.7 (25.60, 31.60)	29.9 (26.00, 32.30)	27.2 (25.10, 31.30)	0.3120^
**Total bilirubin baseline (umol/l), Median (Q1,Q3)**	7.9 (6.20, 11.60)	7.5 (6.00, 11.60)	9.1 (6.90, 11.40)	0.2844^	8.2 (6.80, 11.40)	8.2 (6.30, 12.40)	8.9 (6.90, 10.80)	0.9276^
**Alanine transaminase (ALT) baseline (U/L), Median (Q1,Q3)**	36.0 (23.00, 62.00)	41.5 (28.50, 75.00)	27.0 (17.00, 37.00)	0.0039^	29.0 (18.00, 45.00)	34.0 (21.00, 61.00)	25.5 (17.00, 38.50)	0.2848^
**Aspartate transaminase (AST) baseline (U/L), Mean (SD)**	147.8 (612.75)	139.0 (570.37)	167.7 (709.69)	0.0037^	45.8 (25.22)	52.6 (29.27)	40.0 (19.87)	0.0945*
**Albumin baseline (gm/l), Mean (SD)**	33.1 (4.20)	32.8 (4.24)	33.6 (4.12)	0.4312*	32.0 (3.45)	31.8 (3.67)	32.2 (3.26)	0.6613*
**C-reactive protein (CRP) baseline (mg/l), Median (Q1,Q3)**	116.0 (65.50, 172.00)	124.0 (75.33, 197.00)	77.7 (39.00, 138.00)	0.0377^	123.0 (66.00, 190.55)	144.0 (69.67, 226.00)	91.5 (53.50, 155.50)	0.1595^
**D-dimer Level baseline (mg/l), Median (Q1,Q3)**	1.1 (0.62, 2.42)	1.1 (0.62, 2.51)	1.1 (0.63, 1.85)	0.7959^	1.0 (0.59, 1.85)	0.9 (0.57, 2.25)	1.1 (0.60, 1.85)	0.7844^
**Ferritin Level baseline (ug/l), Median (Q1,Q3)**	421.2 (214.00, 831.40)	440.8 (219.20, 942.95)	399.6 (200.20, 709.10)	0.6360^	407.0 (196.80, 780.00)	425.0 (196.80, 820.10)	399.0 (200.20, 709.10)	0.8327^
**Blood glucose level baseline (mmol/l), Median (Q1,Q3)**	10.5 (7.20, 14.90)	10.2 (7.00, 13.40)	11.4 (7.35, 17.10)	0.1651^	11.4 (7.50, 16.60)	9.9 (6.35, 12.45)	15.5 (7.80, 18.70)	0.0294^
**Lowest PaO**_**2**_**/FiO**_**2**_** ratio within 24 h of admission, Median (Q1,Q3)**	81.1 (63.00, 128.55)	75.1 (61.88, 104.50)	127.1 (71.00, 190.00)	0.0122^	93.8 (66.62, 164.00)	88.5 (63.45, 123.00)	104.2 (70.50, 165.40)	0.3911^
**Lowest MAP baseline (mmHg), Median (Q1,Q3)**	70.0 (63.17, 78.00)	69.0 (63.00, 75.50)	73.5 (64.17, 79.50)	0.3566^	72.0 (66.00, 78.00)	70.0 (66.50, 73.50)	74.0 (65.00, 80.00)	0.3708^
**Pharmacological DVT prophylaxis use during ICU stay, n (%)**	100 ( 97.1)	70 ( 95.9)	30 (100.0)	0.2598**	48 (100.0)	24 (100.0)	24 (100.0)	NC
**Nephrotoxic drugs/material use during ICU stay, n (%)**^**ab**^	97 ( 93.3)	68 ( 91.9)	29 ( 96.7)	0.3786**	44 ( 91.7)	21 ( 87.5)	23 ( 95.8)	0.2963**
**Comorbidity, n (%)**								
**Atrial fibrillation (A Fib)**	4 ( 3.8)	3 ( 4.1)	1 ( 3.3)	0.8625**	1 ( 2.1)	0 ( 0.0)	1 ( 4.2)	0.3122**
**Heart Failure**	15 ( 14.4)	11 ( 14.9)	4 ( 13.3)	0.8404**	4 ( 8.3)	1 ( 4.2)	3 ( 12.5)	0.2963**
**Hypertension**	65 ( 62.5)	50 ( 67.6)	15 ( 50.0)	0.0936^^	25 ( 52.1)	13 ( 54.2)	12 ( 50.0)	0.7726^^
**Diabetes Mellitus**	63 ( 60.6)	47 ( 63.5)	16 ( 53.3)	0.3358^^	26 ( 54.2)	12 ( 50.0)	14 ( 58.3)	0.5623^^
**Dyslipidemia**	31 ( 29.8)	26 ( 35.1)	5 ( 16.7)	0.0621^^	12 ( 25.0)	7 ( 29.2)	5 ( 20.8)	0.5050^^
**Ischemic heart disease (IHD)**	5 ( 4.8)	5 ( 6.8)	0 ( 0.0)	0.1445**	2 ( 4.2)	2 ( 8.3)	0 ( 0.0)	0.1486**
**Chronic kidney disease (CKD)**	4 ( 3.8)	4 ( 5.4)	0 ( 0.0)	0.1941**	2 ( 4.2)	2 ( 8.3)	0 ( 0.0)	0.1486**
**Cancer**	4 ( 3.8)	4 ( 5.4)	0 ( 0.0)	0.1941**	2 ( 4.2)	2 ( 8.3)	0 ( 0.0)	0.1486**
**Deep Vein Thrombosis (DVT)**	0 (0)	0 (0)	0 (0)	NC	0 (0)	0 (0)	0 (0)	NC
**Pulmonary Embolism (PE)**	0 (0)	0 (0)	0 (0)	NC	0 (0)	0 (0)	0 (0)	NC
**Liver disease (any type)**	3 ( 2.9)	1 ( 1.4)	2 ( 6.7)	0.1423**	2 ( 4.2)	0 ( 0.0)	2 ( 8.3)	0.1486**

*DEX* Dexamethasone, *mPRED* Methylprednisolone

^*^T Test / ^ Wilcoxon rank sum test is used to calculate the *P*-value

^^Chi square/ ** Fisher’s Exact teat is used to calculate *P*-value

^ab^Nephrotoxic medications/ material included IV Vancomycin, Gentamicin, Amikacin, Contrast, Colistin, Furosemide, and/or Sulfamethoxazole/trimethoprim

### Mortality

In crude analysis, asthmatic critically ill patients with COVID-19 who received methylprednisolone were associated with higher but insignificant in-hospital mortality compared to dexamethasone (35.0% vs. 18.2%, *P* = 0.22). Moreover, in logistic regression analysis, the odds of in-hospital mortality were higher in the methylprednisolone group compared to dexamethasone; however, the difference was not statistically significant (OR 2.31; CI: 0.56 – 9.59; *P* = 0.25). The 30-day mortality was not statistically significant in crude analysis (31.6% vs. 14.3%, *P* = 0.19) and regression analysis (OR 2.36; CI: 0.51 – 10.86; *P* = 0.27) (Table 2).

**Table 2:** Clinical outcomes of critically ill patients with COVID-19 after PS matching

**Outcomes**	**Number of outcomes/Total number of patients**			**Odds Ratio (OR) (95%CI)**	***P*-value** $
	**DEX** **(*n* = 24)**	**mPRED** **(*n* = 24)**	***P*-value**		
**30-day mortality, n (%)**	3 ( 14.3)	6 ( 31.6)	0.19**	2.36 (0.51,10.86)	0.27
**In-hospital mortality, n (%)**	4 ( 18.2)	7 ( 35.0)	0.22^^	2.31 (0.56,9.59)	0.25
				**beta coefficient (Estimates) (95%CI)**	***P*-value** $*
**Ventilator free days, Median (Q1, Q3)**	20.0 (4.00, 25.00)	15.0 (0.00, 23.00)	0.38^	-0.20 (-0.99,0.60)	0.63
**ICU Length of Stay (Days), Median (Q1,Q3)**	13.0 (6.00, 21.00)	15.5 (9.00, 23.00)	0.53^	0.34 (-0.10,0.77)	0.13
**Hospital Length of Stay (Days), Median (Q1,Q3)**	21.5 (11.50, 29.00)	19.5 (15.50, 29.50)	0.81^	0.06 (-0.35,0.47)	0.76

*DEX* Dexamethasone, *mPRED* Methylprednisolone

^Wilcoxon rank sum test is used to calculate the *P*-value

^^Chi-square test is used to calculate the *P*-value

^$^Logistic regression analysis used to calculate OR and *p*-value

^$*^Generalized linear model is used to calculate estimates and *p*-value

### Ventilator free days (VFDs) and length of stay (LOS)

Patients who received early methylprednisolone had shorter VFDs than the dexamethasone group; however, it did not reach statistical significance (-0.20; CI: -0.99 – 0.60; *P* = 0.63). In addition, the ICU and hospital LOS differences were not statistically significant between the two groups [(0.34; CI: -0.10 – 0.77; *P* = 0.13) and (0.06; CI: -0.35 – 0.47; *P* = 0.76), respectively] (Table 2).

### Complication(s) during stay

Within the first 24 h of ICU admission in non-MV patients, respiratory failure requiring MV was not statistically significant between patients who received early methylprednisolone or dexamethasone (OR = 1.24; 95% CI: 0.06 – 26.78; *P* = 0.89), In addition, AKI and liver injury were not statistically significant [(OR = 1.36; 95% CI: 0.37 – 4.94; *P* = 0.65) and (OR = 0.52; 95% CI: 0.04 – 6.24; *P* = 0.61, respectively)]. The hospital-acquired infections were higher in the methylprednisolone group by twofold compared to patients who received dexamethasone; however, statistical significance was not reached (OR = 2.38; 95% CI: 0.54 – 10.53; *P* = 0.25) (Table 3).

**Table 3:** ICU complications during stay

**Outcomes**	**Number of outcomes/Total number of patients**		***P*-value**	**Odds Ratio (OR) (95%CI)**	***P*-value** $
	**DEX** **(*n* = 75)**	**mPRED** **(*n* = 30)**			
**Respiratory failure requiring MV, n (%)**^b^	1 ( 20.0)	1 ( 25.0)	0.86**	1.24 (0.06,26.78)	0.89
**Acute kidney injury, n(%)**^a^	6 ( 25.0)	7 ( 29.2)	0.75^^	1.36 (0.37,4.94)	0.65
**Liver injury, n(%)**^a^	2 ( 8.3)	1 ( 4.2)	0.55**	0.52 (0.04,6.24)	0.61
**Hospital acquired infection, n(%)**^a^	3 ( 12.5)	6 ( 25.0)	0.27**	2.38 (0.54,10.53)	0.25

*DEX* Dexamethasone, *mPRED* Methylprednisolone

^a^Denominator of the percentage is the total number of patients

^b^Denominator of the percentage is the non-MV patients

**^^**Chi-square test is used to calculate the *P*-value/** Fisher’s Exact teat is used to calculate *P*-value

^$*^Logistic regression is used to calculate the OR and *p*-value

## Discussion

In this multicenter, retrospective study, we aimed to evaluate the use of methylprednisolone and dexamethasone in critically ill asthmatic patients with COVID-19. The use of methylprednisolone was associated with a statistically non-significant higher rate of in-hospital mortality compared to dexamethasone Furthermore, we observed shorter VFDs in patients who received methylprednisolone compared to dexamethasone, but this did not reach statistical significance.

The mortality benefit of dexamethasone has been well-established in COVID-19 by many trials. On the other hand, the benefits of methylprednisolone have been observed in several studies. In the Metcovid trial, methylprednisolone vs placebo in COVID-19 patients was found to have no impact on 28-day mortality [21]. However, patients who were > 60 years of age were found to have a mortality benefit with the use of methylprednisolone as those patients presented with a high CRP level. Interestingly, the study reported a small number of patients known to have asthma. This represented only 3.3% of the methylprednisolone group compared to 1.6% in the placebo group which limits analyzing methylprednisolone mortality benefit in this population.

Similarly, in a systematic review and meta-analysis with 67% heterogeneity, methylprednisolone was associated with 21-day and 28-day mortality reduction compared to no glucocorticoid’s treatment in patients with COVID-19 [22]. Most of the trials in the meta-analysis reported presence of lung disease without specification and only fifty patients were known to have asthma. These observed outcomes correlated with a reduction in inflammatory response with corticosteroids therapy as corticosteroids anti-inflammatory effect may result in a faster recovery from lung damage and a quicker symptom control in COVID-19 which may explain methylprednisolone mortality benefit.

In our study, most of the patients required MV within 24 hours of ICU admission and we observed shorter VFDs with methylprednisolone in comparison to dexamethasone without being a statistically significant finding. In contrast, a prospective triple-blinded randomized controlled trial where they compared the use of methylprednisolone vs dexamethasone for hospitalized COVID-19 patients and found a significantly lower need for mechanical ventilation with the methylprednisolone group [13]. Moreover, Badr et al.did a retrospective single-center study assessing methylprednisolone in COVID-19-associated ARDS and found high VFDs with no impact on mortality [23].

Methylprednisolone has a higher lung tissue concentration-to-plasma and faster onset of action in comparison to dexamethasone which could make it more potent in lung injury [24]. Moreover, plasma inflammatory markers in COVID-19 patients, such as CRP and interleukin-6 (IL-6), have been shown to be significantly lower after methylprednisolone use. However, most of the patients in our study had shorter VFDs with methylprednisolone compared dexamethasone without being a statistically significant. In contrast, a prospective, triple-blinded, randomized controlled trial where they compared the use of methylprednisolone vs dexamethasone for hospitalized COVID-19 patients and found a significantly lower need for mechanical ventilation with the methylprednisolone group [13]. Moreover, Badr et al.did a retrospective, single-center study assessing methylprednisolone in COVID-19-associated ARDS and found high VFDs with no impact on mortality [13, 22–26].

In our study, we found that respiratory failure requiring MV, AKI and liver injury to be not significantly different between those who received methylprednisolone vs dexamethasone. On the other hand, hospital-acquired infections were higher in the methylprednisolone group by twofold compared to patients who received dexamethasone, but this was statistically non-significant and did not affect ICU LOS or hospital LOS. In a systematic review and meta-analysis done by Van Paassen et al., it was observed that secondary infections were reported in those with higher antibiotic and steroids use; however, no data were reported regarding the duration and type of steroids that was associated with secondary infections [10]. Moreover, in a retrospective study assessing secondary infection in severe COVID-19 in patients who received tocilizumab and dexamethasone vs dexamethasone alone. The Tocilizumab group experienced a higher rate of secondary infection. Similarly, in a retrospective single-center study, the combination of methylprednisolone and tocilizumab compared to no methylprednisolone was found to be associated with a higher rate of positive blood cultures [23]. In our study, only nine patients received tocilizumab within 24 hours of ICU admission 5 (20.8%) in the dexamethasone group vs 4 (16.7%) in the methylprednisolone group which may preclude tocilizumab as a major factor in the increase in infections rate observed with methylprednisolone use [27].

Due to the lack of data regarding antibiotic usage and duration in the current study, we were unable to associate a history of antibiotic use to the elevated rate of infection in COVID-19-infected asthmatic patients. Delay in viral clearance, which has rarely been reported in our study, could be a contributing factor. Furthermore, Van Paassen et al. meta-analysis suggested that there might be a delayed viral clearance and an increase in antibiotic use and infection rate related to corticosteroid use which did not result in longer hospital LOS or worsened mortality outcomes. However, four trials included in the analysis found equal viral clearance time with corticosteroids compared to the standard of care. Therefore, delayed viral clearance might be observed in subgroups of patients based on dose, type, and timing of corticosteroid as well as the severity of COVID-19 [10].

Our study has several of advantages, including the use multiple centers and a comparative group. Additionally, we used propensity score matching to reduce bias and create comparable groups.On the other hand, there are various limitations on our study. The relatively small sample size of this study may have made it more difficult to identify a meaningful difference between the treatment groups, which is one of its main drawbacks. Furthermore, as the study was retrospective in nature, its results might be constrained by the presence of residual confounders that weren't taken into account in our PS approach. Additionally, the simultaneous use of antibiotics with established anti-inflammatory properties and inhaled steroids was not examined. 

## Conclusion

The findings of this study suggest that the use of methylprednisolone in asthmatic critically ill patients with COVID-19 may be associated with a non-significant higher rate of in-hospital mortality and shorter ventilation-free days (VFDs) compared to dexamethasone. However, caution should be taken into consideration when interpreting these results, and further studies with larger sample sizes are needed to validate and confirm the findings of our study.

### Supplementary Information

**Additional file 1.** Detailed methods.

## Data Availability

The datasets used and/or analyzed during the current study are available from the corresponding author upon reasonable request.
