# Correction: Evaluation of the use of methylprednisolone and dexamethasone in asthma critically ill patients with COVID-19: a multicenter cohort study

**DOI:** 10.1186/s12890-023-02687-y

**Published:** 2023-10-17

**Authors:** Khalid Al Sulaiman, Ohoud Aljuhani, Ghazwa B. Korayem, Ali F. Altebainawi, Reham Alharbi, Maha Assadoon, Ramesh Vishwakarma, Nadia H. Ismail, Asma A. Alshehri, Faisal E. Al Mutairi, Mashael AlFaifi, Abdullah F. Alharthi, Abeer A. Alenazi, Mai Alalawi, Omar Al Zumai, Hussain Al Haji, Sarah T. Al Dughaish, Abdulrahman S. Alawaji, Haifa A. Alhaidal, Ghassan Al Ghamdi

**Affiliations:** 1 Pharmaceutical Care Department, King Abdulaziz Medical City (KAMC), Riyadh, Saudi Arabia; 2 College of Pharmacy, King Saud Bin Abdulaziz University for Health Sciences (KSAU-HS), Riyadh, Saudi Arabia; 3 King Abdullah International Medical Research Center (KAIMRC), KSAU-HS, PO Box 22490, 11426 Riyadh, Saudi Arabia; 4 Saudi Critical Care Pharmacy Research (SCAPE) Platform, Riyadh, Saudi Arabia; 5 Department of Pharmacy Practice, Faculty of Pharmacy, King Abdulaziz University, Jeddah, Saudi Arabia; 6 Department of Pharmacy Practice, Princess Nourah Bint Abdulrahman University, P.O.Box 84428, 11671 Riyadh, Saudi Arabia; 7 Pharmaceutical Care Services, King Salman Specialist Hospital, Hail Health Cluster, Ministry of Health, Hail, Saudi Arabia; 8 Department of Clinical Pharmacy, College of Pharmacy, University of Hail, Hail, Saudi Arabia; 9 Norwich Medical School, University of East Anglia, Norwich, UK; 10 King Fahad Hospital of the University, AL-Khobar, Saudi Arabia; 11 Department of Clinical Pharmacy Research, Institute of Research and Medical Consultation, Imam Abdulrahman Bin Faisal University, Dammam, Saudi Arabia; 12 Pharmaceutical Care Department, Prince Sultan Military Medical City, Riyadh, Saudi Arabia; 13 Pharmaceutical Services Administration, King Saud Medical City, Riyadh, Saudi Arabia; 14 College of Pharmacy, Shaqra University, Shaqra, Saudi Arabia; 15 Pharmaceutical Care Department, King Abdulaziz Medical City, Jeddah, Saudi Arabia; 16 Respiratory Department Services, King Abdulaziz Medical City (KAMC), Riyadh, Saudi Arabia; 17 College of Applied Medical Sciences, King Saud Bin Abdulaziz University for Health Sciences (KSAU-HS), Riyadh, Saudi Arabia; 18 Pharmaceutical Care Department, King Khalid Eye Specialist Hospital, Riyadh, Saudi Arabia; 19 College of Medicine, King Saud Bin Abdulaziz University for Health Sciences, Riyadh, Saudi Arabia; 20 Intensive Care Department, King Abdulaziz Medical City, Riyadh, Saudi Arabia

**Correction: BMC Pulmonary Medicine 23, 315 (2023)**

**https://doi.org/10.1186/s12890-023-02603-4**

Following publication of the original article [1], the authors flagged the following errors:

1) Affiliations 7, 8, and 11 had been incorrectly detailed.

2) The name of author Ali F. Altebainawi had been miswritten as Ali Altebainawi.

3) Affiliation 8 had been missed from author Ali F. Altebainawi (they are affiliated with affiliations 7 and 8).

The affiliations information has since been corrected in the published article and the corrected affiliations information can be seen in this erratum. The authors thank you for reading and apologize for any inconvenience caused.
