# Supplementary material for: Evaluation of the use of methylprednisolone and dexamethasone in asthma critically ill patients with COVID-19: a multicenter cohort study
Source: BMC Pulm Med. 2023 Aug 28;23:315. doi: 10.1186/s12890-023-02603-4 (PMC10463591; doi:10.1186/s12890-023-02603-4)
Supplement: Supplementary file 1 — Additional file 1. Detailed methods. [file 12890_2023_2603_MOESM1_ESM.docx]

**Supplementary file 1: Detailed methods**

**Study Setting**

The study was conducted at five medical facilities and medical cities in Saudi Arabia with varying geographic distributions. The sites were chosen based on geographic dispersion, availability of electronic data, and willingness to participate. The principal research center was King Abdulaziz Medical City, a tertiary care center in Riyadh. The additional centers included King Abdulaziz Medical City in Jeddah, King Abdulaziz University Hospital in Jeddah, King Abdullah bin Abdulaziz University Hospital in Riyadh, and King Salman Specialist Hospital in Hail.

**Data collection**

Variables and data were collected using the Research Electronic Data Capture (REDCap®) platform hosted by KAIMRC. These variables included the following: demographic data, comorbidities, laboratory, vital signs, baseline severity scores [Acute Physiology and Chronic Health Evaluation II (APACHE II), and Sequential Organ Failure Assessment (SOFA)]. Moreover, other laboratory variables such as renal profile, liver function tests, coagulation profile [e.g. International normalized ratio (INR), activated partial thromboplastin time (aPTT), fibrinogen, and D-dimer], and other markers [e.g. C-reactive protein (CRP), ferritin, and creatine phosphokinase (CPK)] were collected at baseline whenever available. Additionally, acute kidney injury (AKI) status, mechanical ventilation (MV) status, and parameters [e.g. lowest partial pressure of oxygen/fraction of inspired oxygen (FiO2) ratio, and highest FiO2 requirement] within 24 hours of ICU admission were collected. Lastly, early use of tocilizumab therapy and pharmacological VTE prophylaxis were collected.

**Endpoint(s) definition:**

- The 30-day mortality was defined as a death from any cause within the first 30 days of hospitalization. Patients discharged from the hospital alive were presumed to survive.
- AKI was defined as a sudden decrease in renal function within 48 hours, defined by an increase in absolute serum creatinine of at least 26.5 μmol/L (0.3 mg/dL) or by a percentage increase in serum creatinine ≥ 50% (1.5× baseline value) during ICU stay.
- Acute liver injury was defined as an alanine aminotransferase (ALT) exceeding three times the upper limit of normal (55 U/L) or double in patients with an elevated baseline ALT during their ICU stay.

**Detailed statistical analysis**

Continuous variables were reported as a mean with standard deviation (SD), or as a median with lower and upper quartile (Q1, Q3), based on the data distribution. The normality assumptions were assessed for all numerical variables using a statistical test (e.g. Shapiro–Wilk test) and graphical representation (e.g. histograms and Q-Q plots). While categorical variables were reported as crude numbers with percentage.

For 30-day and in-hospital mortality, multivariable Cox proportional hazards regression analysis were utilized. Before fitting the cox model, the proportionality assumption was tested. The assumption was evaluated visually by producing a log(-log) plot and testing the correlation of scaled Schoenfeld residuals with rank-ordered time. For the other outcomes included in this study, multivariable logistic and negative binomial regression analyses were utilized as appropriate. Regression analysis was carried out by considering the PS score as one of the model's covariates. Model fit was evaluated and assessed using the Hosmer-Lemeshow goodness-of-fit test. The hazard ratio (HR), odds ratios (OR), or estimates with 95% confidence intervals (CI) were reported as appropriate. No imputation was made for missing data in this cohort study.
